# RNA-Sequencing Analyses of Small Bacterial RNAs and their Emergence as Virulence Factors in Host-Pathogen Interactions

**DOI:** 10.3390/ijms21051627

**Published:** 2020-02-27

**Authors:** Idrissa Diallo, Patrick Provost

**Affiliations:** CHUQ Research Center/CHUL, Department of Microbiology-Infectious Disease and Immunity, Faculty of Medicine, Université Laval, Quebec, QC G1V 0A6, Canada; Idrissa.Diallo@crchudequebec.ulaval.ca

**Keywords:** Bacterial RNA, high-throughput sequencing, host-pathogen interaction, outer membrane vesicles, small non-coding RNA, CRISPR, virulence

## Abstract

Proteins have long been considered to be the most prominent factors regulating so-called invasive genes involved in host-pathogen interactions. The possible role of small non-coding RNAs (sRNAs), either intracellular, secreted or packaged in outer membrane vesicles (OMVs), remained unclear until recently. The advent of high-throughput RNA-sequencing (RNA-seq) techniques has accelerated sRNA discovery. RNA-seq radically changed the paradigm on bacterial virulence and pathogenicity to the point that sRNAs are emerging as an important, distinct class of virulence factors in both gram-positive and gram-negative bacteria. The potential of OMVs, as protectors and carriers of these functional, gene regulatory sRNAs between cells, has also provided an additional layer of complexity to the dynamic host-pathogen relationship. Using a non-exhaustive approach and through examples, this review aims to discuss the involvement of sRNAs, either free or loaded in OMVs, in the mechanisms of virulence and pathogenicity during bacterial infection. We provide a brief overview of sRNA origin and importance and describe the classical and more recent methods of identification that have enabled their discovery, with an emphasis on the theoretical lower limit of RNA sizes considered for RNA sequencing and bioinformatics analyses.

## 1. Introduction

More than 60 years ago, RNA was already mentioned as a critical player in the regulation of gene expression [1]. According to the central dogma of biology, RNA was considered as “simple” macromolecule carriers of genetic information and used to generate proteins. It was in the 1980s that the concept of regulatory RNA was introduced [2,3], with the gradual discovery of several types of RNA species that do not code for proteins (hence their name, non-coding RNAs) but still play critical roles in many biological processes.

Bacterial small regulatory RNAs (sRNAs), which are the subject of this review, were characterized in prokaryotes [4,5,6] well before the discovery of the very first microRNAs (miRNAs) and small interfering RNAs (siRNAs) in eukaryotes.

Major high-throughput technological advances have allowed the emergence of sRNAs, which now represent the most important post-transcriptional class of regulators among prokaryotes. Similar to miRNAs, their functional analogs in eukaryotes, most sRNAs have multiple targets and (trans-) acts by base-pairing with target messenger RNAs (mRNAs). sRNAs can regulate their targets in *cis* as well [7]. Such targets are not invariably RNAs, and include proteins (e.g., CsrB, 6S) [8,9] and DNA [10,11]. sRNAs may thus regulate several aspects of gene expression, such as transcription, translation, mRNA stability, and DNA maintenance or silencing [3].

sRNAs are between 50 and 200 nucleotides (nt) in length and are generally expressed under specific conditions. Often, their primary function involves inhibiting translation and destabilization of mRNAs [12]. From a mechanistic angle, *trans*-encoded sRNAs base pair in anti-sense orientation with complementary sequences on the mRNA target, often in the vicinity of the translation initiation region, but also, to a lesser extent, in the coding sequence or the 3’ untranslated region (UTR). In most cases, this interaction leads to the co-degradation of base-paired RNAs (sRNA-mRNA). It involves ribonucleases, such as RNase E, but also Hfq (host factor for bacteriophage Qβ) RNA chaperone, which mediates and stabilizes RNA-RNA interactions [13].

In bacteria, mRNA degradation modulates protein biosynthesis and thus allows their rapid adaptation to environmental changes, which makes mRNA stability an essential factor in differential gene expression. mRNAs have a relatively short half-life, ranging from 30 s to 20 min [14,15], which allows rapid modification and adaptation of the bacterium’s gene expression profile in response to specific environmental cues [16,17,18,19]. High-throughput RNA-seq approaches are now unveiling that this adaptive capacity, which is under the control of sRNAs, is part of the virulence.

### 1.1. sRNAs Origins and Evolution

sRNAs are heterogeneous in size and structure, and have diverse sources [3,20]. Typically encoded in the intergenic regions (IGRs) of bacterial genomes [21], some of the sRNAs are primarily conserved in bacteria, while others are characteristic of a given species [22]. Generally, they exhibit essential sequence divergence. In addition to IGRs, sRNAs may also be generated from many other sources [23,24,25,26,27,28]. It is challenging, however, to discuss hallmarks for sRNAs because they have so little in common in terms of their origin in the genome, their transcript length, or their structure [29].

The diversity of sRNAs is credited to duplication [30], deletion [31], or horizontal acquisition [32]. Another potential source of sRNAs is the genomic rearrangements of IGRs, which may also increase disparities [22].

In many respects, the relatively simple operating mechanism of sRNAs accounts for their variety and rapid evolution [33,34]. The dynamics around the origin, evolution, and loss of sRNAs have been uncovered by previous studies and reviewed recently [35].

Other sources of sRNAs, such as transcriptional noise and exaptation, are also considered [36]. In perspective, these authors suggest the use of molecular archeology, synthetic biology, and experimental evolution to understand better the processes underlying the generation of functional sRNAs.

### 1.2. sRNAs Relevance

The analogy made with miRNAs underlies the relative importance of sRNAs, which are key players in the control of gene expression in bacteria. In many respects, sRNAs are much more effective than regulation via transcription factors or protein-based mechanisms [20,37,38].

The use of sRNAs, to regulate the expression of its genes, is advantageous for bacteria in many ways: reduced metabolic cost, celerity of action, integration into a simple, precise, and controlled gene regulatory network, ease of vesicular packaging and transfer to other cells, preserved function in different cells, and subsequent recycling [33].

Specifically under stress conditions, sRNAs actively participate in the rapid reprogramming of cell metabolism by regulating the expression and stability of several target mRNAs [12]. The ability to sense environmental changes so quickly is also important, through the regulation of virulence genes, during infection of the host where the micro-environment is variable and inhospitable.

These long-neglected sRNAs are now recognized as playing a significant physiological role in bacterial metabolism [39,40] and bacterial virulence [41,42]. The crucial role of sRNAs in the post-transcriptional regulation of gene expression and their involvement in host-pathogen interactions make them prime targets in the search for new antibiotics. Indeed, the most recent class of antibiotics against gram-negative bacteria were developped in 1960 and, today, the increase in antibiotic resistance is one of the major challenges of our era. The potential value of sRNAs as a therapeutic target for the treatment of multidrug-resistant bacterial infection is currently being addressed (For review, see [43]), and trials are at their very beginning.

From another perspective, the crucial role of sRNAs is also illustrated through the genetic approach of RNA interference (RNAi) and the large diversity of genome-scale editing tools (for reviews [44,45]), such as Clustered Regularly Interspaced Short Palindromic Repeat (CRISPR) system. Originally a natural system dedicated to genome editing, as part of the bacterial adaptive immune response system, CRISPR has become the swiss army knife [46] of genetics, with promises and challenges [47] in therapy and biotechnology.

The system is based on a complex formed by a short non coding single-guide RNA (sgRNA) and a nuclease CRISPR-associated (cas) protein. It has been adapted in several modules to specifically intervene on gene expression. Thus, CRISPR interference (CRISPRi), similar to RNAi in concept but more robust in performance, uses a sgRNA and a version of the cas9 protein devoid of endonucleolytic activity (deadcas9 or dcas9) to mediate gene silencing [48]. Based on the same idea, CRISPR activation (CRISPRa) uses a dcas9, but is designed to activate transcription [49].

## 2. sRNAs Identification

Although several approaches and strategies have been developed to identify sRNAs, most of them have become obsolete or somewhat peripheral, with the introduction of advanced sequencing technologies.

### 2.1. The Classical Methods up to the Post-Genomic era

The strategies and techniques used over the years to identify sRNAs, from the 70s to the post-genomic era of the 2000s, were critically summarized fifteen years ago [50].

One of the pioneering approaches they described is RNA labeling and staining. This method does not require prior knowledge of sRNA characteristics and usually involves radioactive labeling, 1 or 2D-PAGE autoradiography followed by fingerprinting of selected RNA bands [2]. The limitation of RNA labeling and staining is that it fails to distinguish sRNA sequences and abundant, processed fragments of ribosomal RNAs (rRNAs) or transfer RNAs (tRNAs).

sRNAs can be identified by genetic screens using tools such as a genomic multicopy library, translational fusion with lacZ, and cloning into plasmids or phages. Genetic screens are practical methods for functional studies but particularly complicated if the sRNA is either essential or toxic when overexpressed.

One of the other classic techniques used is copurification with proteins that are limited to a subclass of sRNAs and requiring highly specific antibodies. The Hfq is commonly used because it binds sRNAs [3,51,52] to ensure stability and enhanced interaction with the mRNA target. Therefore, antibodies directed against Hfq are beneficial for such experiments.

The increasing availability of complete genome sequences of bacteria has gradually highlighted in silico approaches for the search and identification of sRNAs. However, in contrast to the RNA labeling and staining methods aforementioned, biocomputational searches require knowledge of the characteristics of sRNAs.

Microarray detection is also a method of choice for sRNAs expression studies or even discoveries. It is suitable for the detection of species-specific sRNA transcripts yet still generally requires coverage of IGRs (major providers of sRNAs). Shotgun cloning or Sanger sequencing of size-fractionated RNA also remains reliable alternatives for the study and discovery of sRNAs, even if they are laborious.

These techniques are not secluded and are rather complementary. The advent of new modern technologies may have taken their spotlight, but not their value, as they often serve as complementary validation approaches.

### 2.2. HTS-Based Approaches and the Dogma Regarding the Lower-End Cutoff of RNA Size

These are the so-called next-generation sequencing techniques, of which RNA-seq is one of the cornerstones for the identification of sRNAs. After RNA isolation, the library preparation includes various steps, some of which are subject to the discretion of the experimenter.

To improve the sensitivity, the very abundant rRNAs (and their fragments) are often depleted, and depending on the purpose, mRNAs or specific RNAs may also be fished out by sequence-specific hybridization probes. The study of sRNAs and some families of small RNAs of discrete length, such as miRNAs, usually involves a size-fractionation step on a gel, from which they are extracted using a suitable kit. Once the RNAs have been isolated based on their length, they are linked to 5’ and 3’ adapters and reverse transcribed into cDNAs before being subjected to sequencing.

In this postgenomic era, the number of bacterial sRNAs is continuously growing, thanks to modern techniques that interrogate entire transcriptomes [53]. Developments in high-throughput RNA-seq–based approaches, with relevance to sRNA biology, have been discussed recently [54]. Consequently, we will focus less on detailing the features of RNA-seq for their use in the identification of sRNAs, and instead, turn our attention to a dogma regarding the choice of the sequencing RNA size window.

The commonly used standard protocol of sequencing platforms or genomics facilities set the lower-end cutoff of RNA sizes to 15 or 16 nt [55], with the premise that RNAs shorter than this size diminish the sensitivity, are degradation products or too small to have any biological significance.

Even when they are detected by sequencing, RNAs shorter than 15 or 16 nt are generally readily excluded from further bioinformatics analyses and databases. Moreover, it is hard to discriminate degradation fragments that occurred after RNA isolation from those produced in the cell.

Contrary to what we expected, transfer RNA-related fragments (tRFs) are not merely by-products of tRNA random degradation, but rather functional molecules with precise and important roles in the regulation of gene expression [56]. rRNA fragments (rRFs) are also currently under similar scrutiny and are actively being investigated to determine their functional activities, as reviewed recently [57].

Detailed characterization of sRNAs has ultimately given them an average size ranging from 50 to 200 nt, even if functional sRNAs shorter than 50 nt have already been reported [58]. sRNAs smaller than 100 nt are the most abundant, followed by those between 100 and 200 nt [59]. By accepting the idea that some sRNAs may be functional analogues of miRNAs, which are generally between 18 and 24 nt in length, it may be appropriate to reconsider the window of bacterial RNA sizes of interest in order to enhance the possibility of finding sRNAs shorter than 50 nt, the size of miRNAs and perhaps even shorter.

Similar to miRNAs, sRNAs base-pair imperfectly to their RNA target through a relatively short region. Even if the potential pairing region is vast (10 to 25 nt), only a few nucleotides appear to be required and critical for the regulation [60,61]. For instance, the seed region of miRNAs, base-pairing with their mRNA targets, involves the first 8 or 9 nt. These observations suggest that sRNAs shorter than 15 nt may bear functional significance and may have escaped detection because of the generally accepted use of sequencing protocols standardized on a dogma.

In addition, as with their miRNA analogues, sRNAs can be found in functional ribonucleoprotein complexes [62]; particularly, since their targeted messengers can couple simultaneous transcription and translation [63]. It is acknowledged that miRNAs (18–24nt) function through miRNA-RNA-induced silencing complex (RISC) complex [64] that does not exist in bacteria.

Despite being quite different in many respects, bacterial Hfq performs the tasks of stabilization and presentation of sRNAs similar to RISC [65]. As such, the resulting complexes can be an important niche of interest for the identification of sRNAs. This is further illustrated by the use of co-precipitation techniques with Hfq (see Section 2.1).

Complexes formed by proteins belonging to the class of RNA chaperone, scaffolding, enzymatic [62], CRISPR associated [66] and those associated with the degradosome [67] can also be interesting targets for High Throughput Sequencing (HTS) assays, if we consider the possible existence of very small sRNAs of the miRNA class.

Due to imprecise gel cutting and fractionation of RNA species between 16 and 30 nt in length, Plante et al. [68] made the serendipitous discovery of a 12-nt endogenous RNA species corresponding to the 5′ half of the microRNA let-7, which was termed semi-microRNA (smiRNA). Devoid of any direct mRNA regulatory activity, a 12-nt smiRNA sequence could modulate the ability of the miRNA from which it derives to mediate translational repression or cleavage of an mRNA target, supporting the functional significance of an RNA species half the length of miRNAs.

Arbitrary cut-offs have long hampered the discovery of miRNAs, which were, at the time, considered as mere RNA degradation products, too small to be either functional or meaningful [57]. It has been demonstrated that the high efficiency of the CRISPR/cas system is intimately linked to the length [69] of sgRNA (often set to 20 nt), and that, contrary to common misconception, the extension of the guide sequence does not improve targeting specificity [70].

Therefore, it would be interesting to apply a new, more permissive cut-off length in order to expand the repertoire and study of sRNAs, as well as to a context where the minimal length of miRNAs (17–24 nt) and tRFs; (16–40 nt), which are recognized as major actors in gene regulation [71,72], corresponds to the lower limit of the standard window of RNA sizes (15 or 16 nt).

As a result, the window of RNA sizes under analysis represents an additional parameter to consider, in addition to the experimental and analytical concerns reported to date on RNA sequencing technologies [54,73]. We can thus more adequately embrace the third revolution in sequencing technology [74].

## 3. sRNAs in Host-Pathogen Interactions

Whereas the involvement of sRNAs in post-transcriptional gene regulation is well established and described (see Introduction section), their role in virulence and pathogenicity during infection and adaptation in superior hosts as a distinct class of virulence factors, is less well documented.

sRNAs are major players in the bacterial subterfuge to survive under extreme stress conditions, both within and outside a host [3,75]. This adaptive capacity, which is partly based on the rapid and efficient regulation of sRNAs, appears to be the first pillar of virulence as well as the persistence of bacteria through time.

High-throughput RNA-seq has revealed that many bacteria, in addition to the mechanisms involving known traditional virulence factors, widely use sRNAs to conquer its host, destroy its defenses, and finally control long-term infection. sRNAs may act either as virulence factors or as regulators of virulence factors.

### 3.1. sRNAs, A Distinct Class of Virulence Factors

The ability to rapidly adapt to a hostile and shifting environment is key to defining the role and importance of bacteria in host-pathogen interactions. Although the focus had long been on proteins, as the main contributors to this adaptive capacity, it is now clear that RNAs, particularly sRNAs, have emerged and acted as significant regulators of adaptive responses [76,77,78]. In this section, we will explore and illustrate through examples the crucial role of sRNAs in the control of pathogenesis of some gram-negative and gram-positive bacterial strains.

#### 3.1.1. Gram-Negative Bacteria

*Edwardsiella tarda.* With a wide range of hosts (fish, human, animal), *Edwardsiella tarda* is an opportunistic bacterium from the enterobacteria family, causing intestinal and extra-intestinal infections in humans, mainly in people with weakened immune systems [79]. The ability to survive and replicate in various host cells may depend on sRNAs, as recently demonstrated. With the use of RNA-seq, Gao et al. [80] have identified a dozen regulatory sRNAs, including EsR240. This 596-nt sRNA regulates a battery of genes linked to metabolic functions and ion transport. It allows *E. tarda* (ET13 strain) to adapt to the severe environmental conditions of the host and to establish long-term infection. In teleosts, it has been reported that *E. tarda* prevents cell apoptosis to ensure its survival by promoting the expression of anti-apoptotic genes in the host [81]. The mechanisms underlying this process remain to be determined. However, such a strategy for intracellular survival may involve the regulatory network of sRNAs. A few years earlier, the inventory of virulence mechanisms in this bacterium focused on exoenzyme and secretion systems (SS) primarily type 3 (T3SS) and 6 (T6SS) as major elements [82].

*Yersinia pestis.* In bacterial species, such as *Yersinia pestis* (the infectious agent of bubonic plague), the T3SS is a significant virulence factor that is required to deliver mostly bacterial protein effectors to the host [83]. Functional studies on YsR40 sRNA (362 nt in length) have shown that its knock-down reduces infection efficiency in host cell culture and bacterial cell growth in response to stress [84]. Similar findings were obtained in a yersiniosis mouse model infected with *Y. pseudotuberculosis* and in which multiple sRNAs were mutated [85]. More than 180 sRNAs, including 37 new ones, were discovered upon deep RNA-seq of the *Y. pestis* transcriptome and sequence analysis of sRNA candidates [84]. A more prominent role for sRNAs in virulence is to be expected.

*Salmonella.* In the same vein, *Salmonella* invasion of epithelial cells or macrophages is reduced upon deletion of InvS sRNA (89 nt) [86] and isrM sRNA (329 nt) [87], which respectively controls the level of certain T3SS apparatus proteins (PrgH) or flagellar gene expression (*fimZ*) and some pathogenicity islands effector/regulator (SopA, HilE). In an in vitro non-proliferative state, *Salmonella*-specific sRNA RyhB-2 (80 nt), acting in synergy with RyhB-1, contributes to attenuate intracellular bacterial growth and several other sRNAs appear to be actively involved throughout the intracellular infection process [88]. *Salmonella* adapts very well to the host environment, likely due to the induction of sRNAs encoded within genetic islands, which may contribute significantly to its virulence [89]. A catalog of *Salmonella* sRNAs has been established [90].

*Brucella melitensis.* Studies on another bacterial strain, *Brucella melitensis*, provided evidence that Bsr1141 (75 nt), an abundant Hfq-dependent sRNA, modulates the expression of virB2 gene, which is one of the 12 genes (*virB1*–*12*) of the VirB type IV secretion (T4SS) of *Brucella.* Thanks to Bsr1141 Brucella could withstand different environmental conditions during their long-term residence in host macrophages [91]. The major virulence factors reported in *Brucella* pathogenesis are often lipopolysaccharide (LPS), T4SS, and the BvrR/BvrS system [92,93]. sRNAs are rarely or never mentioned as virulence factors. It may be relevant to consider the regulatory network and mechanisms of action of sRNAs in the quest for a druggable therapeutic target against the febrile disease of human brucellosis.

*Coxiella burnetti.* As an obligate intracellular bacterial pathogen, *Coxiella burnetti* has evolved strategies to circumvent the host’s defenses, in which sRNAs may be involved. The zoonotic agent of Q fever was investigated by in-depth RNA-seq analysis, which unveiled the existence of about fifteen new sRNAs ranging between 99 to 309 nt in length. Some of these sRNAs seem to have roles in regulating bacterial response related to intracellular growth and survival. Before this study, no sRNA was directly identified as being implicated in the virulence of *C. burnetii* [94]. In a recent study performed by the same group, functional *C. burnetii* small RNA 12 (CbsR12) has been characterized. CbsR12 is involved in the regulation of the methionine cycle, pyrimidine biosynthesis and *Coxiella* vacuolar protein D (CvpD) [95], a protein acting as a crucial component in parasitophorous vacuole generation (required for successful intracellular parasitism) [96].

*Escherichia coli.* The well-known laboratory strain *Escherichia coli* is a member of the complex ecosystem intestinal microflora of humans and other mammals. *E. coli* is the predominant non-pathogenic facultative flora of the human intestine; however, some of its species contain many pathovars that cause a variety of diseases associated with morbidity and mortality worldwide [97,98]. Collectively referred to as diarrheagenic *E. coli*, the six most prominent pathovars (pathotypes) are enterohemorrhagic *E. coli* (EHEC), enterotoxigenic *E. coli* (ETEC), enteropathogenic *E. coli* (EPEC), enteroaggregative *E. coli* (EAEC), enteroinvasive *E. coli* (EIEC), and diffusely adherent *E. coli* (DAEC) [99].

The EHEC and EPEC *E. coli* pathovars are two of the greatest threats to public health. EHEC and EPEC cause intestinal lesions widely documented under the name of attachment/effacement A/E lesions. The locus of enterocyte effacement (LEE) is a pathogenicity island (PAI) that encodes a T3SS, chaperones, and effector proteins [100,101,102]. The sRNAs regulatory role in relation to the LEE of the EPEC and EHEC has been reviewed recently [103]. The authors argue that more than 40 protein virulence factors associated with LEE have been identified and characterized, while sRNAs are just being unveiled. Moreover, for all the pathovars mentioned above, no sRNA is clearly cited as an actor or virulence factor but mainly effector proteins [99].

#### 3.1.2. Gram-Positive Bacteria

Although sRNAs remain relatively poorly studied in gram-positive bacteria, they are still relevant in the virulence and pathogenesis of these taxa. In this sub-section, we will focus on *Staphylococcus aureus*.

*Staphylococcus aureus.* This opportunistic human pathogenic bacterial strain is a significant public health problem [104], notably with the emergence of the community-associated methicillin-resistant *Staphylococcus aureus* (CA-MRSA). Its capacity to evade and/or neutralize innate host defense mechanisms is based on a range of virulence factors [105,106], including the phenol-soluble modulins (PSMs), which are cytolytic toxins that kill white blood cells [107,108].

Carried by all species of *Staphylococcus*, the pore-forming PSMs strongly influence the virulence of CA-MRSA. The αPSM transcript is subjected to sRNA-mediated regulation. In a murine abscess model, sRNA Teg41 was identified as being required for virulence. Teg41 (200 nt) positively influences αPSM production and, therefore, the hemolytic activity of *S. aureus* [109]. A *Staphylococcus* strain carrying a mutated Teg49 sRNA exhibited a reduced bacterial load compared to the wild-type *S. aureus* strain. Comparative RNA-seq data also revealed putative virulence targets that Teg49 could regulate [110].

sRNAs have been poorly studied in gram-positive bacteria, compared to gram negatives, [111], and have rarely been directly cited as virulence factors; however, in *S. aureus*, they have regained a certain momentum in the quest for virulence factors. The regulatory RNA molecule *S. aureus* II (encoded by the agr locus) is a good example. RNAIII regulates the synthesis of a major pleiotropic transcription factor and a broad set of virulence and other accessory genes [77,112,113,114]. *S. aureus* RNAIII is one of the longest regulatory RNAs that control several virulence genes encoding exoproteins and cell-wall-associated proteins.

The rich and varied repertoire of sRNAs gives *S. aureus* many pathogenic properties that facilitate implantation and integration in the hosts [115,116].

The involvement of sRNAs in virulence and pathogenesis was also shown in other gram-positive microorganisms, such as *Streptococcus* [117,118,119,120,121,122]*, Listeria* [123,124,125,126,127], *Enterococcus faecalis* [128], and Clostridium [128,129].

Regarding virulence and pathogenicity, studies have traditionally focused on protein functions rather than sRNA-mediated regulatory mechanisms. The emergence of high-throughput sequencing technologies may help uncover and define the role of sRNAs in multiple mechanisms of virulence and pathogenicity and establish sRNAs as a distinct class of virulence factors that are important for bacterial infection.

### 3.2. OMVs, Emerging Carriers of sRNAs

Pathogenic and non-pathogenic bacteria produce outer membrane vesicles (OMVs) under stress or normal growth conditions (for reviews, see [130,131]. Bacterial OMVs are involved in adaptation (response to stress), survival (nutrient acquisition), and cell-to-cell communication (biofilm, quorum sensing). In addition, they contribute to the promotion of pathogenesis through the delivery of virulence factors [132]. While type III, type IV, and type VI secretion systems require close physical contact between cells, OMVs represent a vehicle capable of delivering bioactive molecules, such as sRNAs, from bacteria to other bacteria or host cells. Artfully referred to as secretion system type zero, OMVs offer many advantages for the transport of biomolecules (for review, see [133]).

The cargo of OMVs consists of different types of macromolecules, such as lipids, proteins, and nucleic acids. In contrast to vesicles released by eukaryotic cells [134,135,136,137], the RNA content of bacterial OMVs has only been investigated recently [138,139].

OMVs derived from a uropathogenic strain of *E. coli* (UPEC) contain a wide variety of RNA species, such as rRNA, tRNA, and mRNA [140]. Beyond their profile, little is known about their biological role once delivered to host cells via OMVs. It has been shown, however, that several classes of RNAs (e.g., mRNA, sRNA, miRNA) secreted through vesicles can have functional activities in their recipient cells [135,141,142].

Since there are only a few studies that have focused on the RNA content of bacterial OMVs, the potential role of vesicular, bacterial sRNAs in host-pathogen interactions remains to be explored [143].

The latest studies have shown that *P. aeruginosa* can modulate the host’s immune response through sRNAs contained in its OMVs [58], in one of the first demonstrations of the trans-kingdom biological activity of a regulatory sRNA contained in bacterial OMVs.

Another study on *Salmonella* highlighted the differential RNA loading into OMVs depending on environmental or growth conditions. Some RNAs might be specifically loaded or enriched into OMVs, suggesting a functional role in target cells [143]. There was no indication of the presence of RNA chaperone Hfq in the OMVs. However, under certain growth conditions, it is reported that some Hfq-dependent trans-acting sRNAs involved in virulence are enriched in salmonella enterica OMVs [143].

Research has shown that OMVs play a critical role in cell-to-cell (or bacterial-host) interactions through the variety of its inner and outer composition, often with a focus on proteins. sRNAs have been associated with the biogenesis and production of OMVs but not defined as specific components of these OMVs [144,145]. The progressive discovery of sRNAs in OMVs [58,138,139,146] is expected to lead to further investigations on the evolutionary and strategic advantage of delivering sRNAs through OMVs in host-pathogen interactions. Coupled to their immunomodulatory properties [147], OMVs may represent a vehicle of choice for the transport of sRNAs targeting specific genes in the host. These multiple factors and mechanisms remain complex and challenging to study but open a new paradigm on host-pathogen dynamics.

## 4. Conclusions

**Figure 1:**
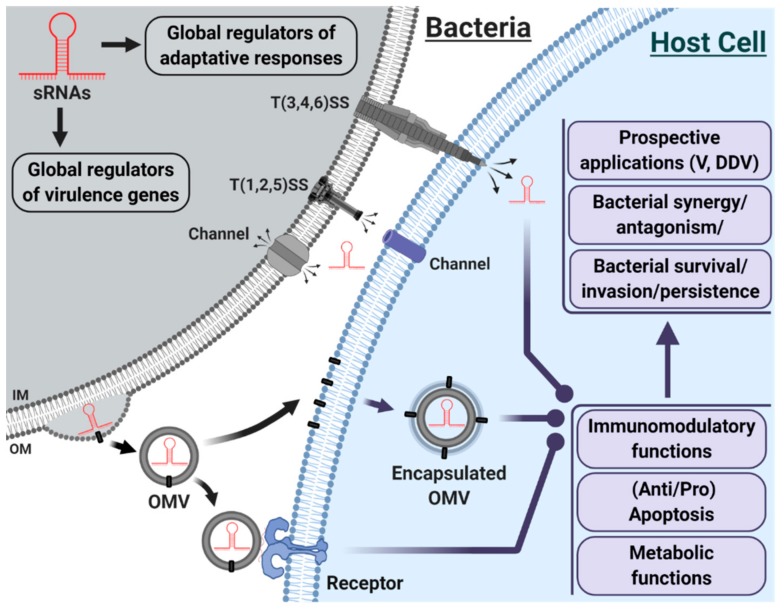
Schematic representation of bacterial host-pathogen interactions, with an emphasis on sRNAs.

sRNAs play a central role in an impressive gene expression regulatory network and are involved in diverse biological processes (Figure 1). Yet, the role and function of bacterial sRNAs, especially those delivered through OMVs, in host cells remain to be demystified.

The routine use of high-throughput RNA-seq has led to significant advances in the field of sRNAs. The experimental and analytical concerns for this method, including the dogmatic lower limit of the RNA sequencing window, must be addressed to allow the full exploitation of its potential for breakthroughs. As sRNAs have just begun to unravel their secrets, we are learning more about their origin, their extraordinary diversity, their evolution and, most importantly, their functions.

The emergence of OMVs and of their bioactive sRNA content may soon call for a paradigm shift in host-pathogen interactions, which may not be defined solely by proteins and outer membrane contacts.

In host-pathogen interactions, the inventory of virulence factors [T(1,2,3,4,5,6)SS; exoenzymes; other proteins] rarely included sRNAs. The development of HTS repositioned sRNAs at the heart of gene regulation through their unique properties; they are increasingly recognized as a distinct class of virulence factors. sRNAs are effective in the regulation of virulence genes and adaptive responses within the bacterium. They may also be transferred, via different mechanisms, to host cells, where sRNAs may affect the immunomodulatory, metabolic, and apoptotic functions of the cell, with multiple consequences (synergy, antagonism, persistence, invasion, etc.). sRNAs may be delivered to the host through injection via T(3,4,6)SS, secretion via channels or packaging in OMVs. IM = Inner Membrane; OM = Outer Membrane; OMV = Outer Membrane Vesicle; T(1,2,3,4,5,6)SS = Type (1,2,3,4,5,6) Secretion System; V= Vaccines; DDV = Drug Delivery Vehicles. Original figure created with BioRender.com and inspired by the references [58,132,133], but not drawn to scale.
